# Environmental impacts analysis of Moroccan olive cake combustion in Stirling motor cogeneration system: Case study

**DOI:** 10.1016/j.heliyon.2024.e28516

**Published:** 2024-04-04

**Authors:** N. Rassai, N. Boutammachte

**Affiliations:** a Advanced Systems -Engineering Laboratory-National School of Applied Science, Ibn Tofail University, University Campus, Po Box 242, Kenitra, Morocco; b 2ER Team-Department of Energy-National School of Arts and Crafts, Moulay Ismaïl University, Meknes, Morocco

**Keywords:** Olive cake, Pulverized, Combustion, Excess air ratio, Stirling motor

## Abstract

Morocco is the world's 5th largest olive oil producer with 160,000 tons in 2020/2021 and the production of 1 L of olive oil generated approximately 5 kilos of waste olive cake, this type of waste is dangerous for the environment and especially for groundwater. To address this issue and take into account the thermal properties of olive waste, our project's goal is to produce power and heat by using solid olive waste in a Stirling cogeneration system. Therefore, the goal of this article is to examine by using CFD tools the environmental impacts of the Moroccan solid olive waste burning in a CHP (combined heat and power) unit. The idea is to integrate a smart renewable energy system based on olive cake and the Stirling motor to produce electricity in order to employ this electrical energy for water pumping in rural areas.

The impact of the excessive air ratio on temperature profile and exhaust has been investigated and discussed. In addition, the pollutant species in air has been presented, and compared with guideline values given by some international organization for health and environment. Results show that the highest excess air ratio gives high temperatures with a homogeneous distribution around the Stirling motor hot heat exchanger, and minimum emissions of CO, CO_2_, NO, NO_2_ and NH_3_ that do not exceed the values set by WHO, OSHA, and NIOSH.

## Nomenclature

C_D_: The drag coefficient [dimensionless]
CFD: Computational Fluid Dynamics
Dp: The diameter of particle [m]
OC: Olive Cake
R_ep_: The relative Reynolds number [dimensionless]
t: Time [s]
T: Mean temperature [K]
u: Instantaneous velocity [m/s]
u_p_: The particle velocity [m/s]
V: Volume [m^3^]
μ: Fluid dynamic viscosity [Pas]
λ: Excess air ratio [dimensionless]
ρ: The density of fluid [kgm−3]
ρ_p_: The density of particle [kgm−3]
Φ: Char combustion stoichiometric ratio [dimensionless]

## 1 Introduction

In Morocco, the irrigation of an area of 30 ha cultivated with vegetables using a 180 m well requires 52.6 tons of butane gas annually, the equivalent of 4380 of the 12 kg gas bottles [1]. And with the expansion of agricultural activities, the energy demand will be increased and establish energy limitations to Moroccan farmers. For this reason and others, Morocco has a plan to have 19% of biomass power as a source of energy by 2040, generating heat and electricity. One of the most important biomasses in Morocco is olive waste (about 1–2 million tons produced in 2020 [2]).

Nevertheless, the recycling of olive waste in Morocco is very limited; few projects are carried out by national and international companies to generate heat and electricity including Lesieur Cristal which ensures 50% of its heating needs from 2015 by installing two boilers that work with olive cake [3]. In 2012, Cosumar also installed a biomass boiler powered by olive cake in its Zaio plant in the Oriental region, which allows it to reduce its energy consumption by 25% between 2013 and 2020 [4]. In addition, to remain competitive, Lafarge company has been committed to the recovery of alternative fuels, through the import of shredded tyres and the use of olive cake since 2004 [5]. In 2014, Veolia commissioned three Vyncke biomass boilers for Renault Tanger with a total capacity of 18.5 MW to cover the plant's thermal needs, namely the heating of cabins, bathrooms, and paint ovens; the thermal process of the entire plant and the heating of certain workshops in winter. 'These boilers can be supplied with both fuels olive cake and wood, depending upon the availability of the material [6]. In the context of the Meknes-Jaen/Spain agreement, Agro-pôle olive Meknes has launched The Olea Green Meknes project; it is one of the innovative renewable energy projects to be developed in Meknes and its region, for the valorization of olive waste (solid-fluid), the improvement of the competitiveness of the Moroccan olive oil sector and above all for a sustainable olive growing that respects the environment [7]. Another important company named Aveo Energy produces no less than 70,000 tons of biomass per year from the olive cake and other agricultural waste to cover thermal needs to diverse client especially hotels [8].

For this reason, the idea of our project is not to produce just heat from Moroccan olive cake (OC) but also electricity by using the Stirling motor (SE); the hot heat exchanger can be heated by the flame, as when using a liquid fuel or a gas fired burner. However, selection of adequate settings for the combustion process is quite difficult because of the olive cake's heterogeneous constitution, pollutant emissions and deposition of ash. To fully comprehend the combustion process of olive cake, it is imperative to use the current computational fluid dynamics (CFD) methods to investigate the effects of combustion parameters, such as particle size and surplus air ratio, on various features.

Moroccan olive cake contains a lot of qualities, including a high calorific value (HCV) of almost 22,000 kJ/kg and a high abundance throughout various regions (roughly 2 million in 2020). [9], very cheap and free of sulfur [10]. Because of all these benefits, the OC is a good alternative to fuels. There are numerous advantages to using olive cake as fuel for a Stirling engine. But, like other types of wastes, Olive Cake combustion can create problems if suitable and appropriate measures are not considered during the combustion process; because it can be source of many pollutant emissions and affects the operation and maintenance of SE ([11,12]). Some of these considerations are the excess air coefficient and the size of the olive cake particle. In order to understand the phenomena taking place in solid biomass combustion, It is essential to investigate how these factors affect pollution, temperature, char deposition, and the upkeep and functioning of SE using computational fluid dynamics models.

Our comprehension of the combustion mechanism can be enhanced by the use of numerical simulation. The process of burning particles and gases, as well as the factors influencing temperature, velocity, and pollutant emissions, may all be clearly outlined by it. In actuality, no prior CFD studies have been published in the literature that have looked into using olive cake as a fuel for the Stirling device; most of the studies are focused on wood combustion. Various CFD codes have been used to model the combustion of biomass by different methods; depending on the type of the combustor (pulverized fuel, fluidized, packed) and on the particle model; Mehrabien et al.([13,14]) studied the packed bed combustion, while Singh et al. [15]examined the fluidized bed solid combustion process; they established an outline of the different technologies of modeling used on the wood burning process that presents several numerical models required in a computational model of combustion according to the Eulerian-Lagrangian or Eulerian-Eulerian approach. In a pulverized fuel-focused paper, Bonefacic et al. [16] listed computational techniques for co-combustion of biomass and pulverized coal in a vertical cylindrical combustor (20 kW). Later, in a vertical combustor, Elorf et al. [17] investigated the impact of turbulent flow on the dynamic of the pulverized OC flame.

An external combustion motor, also known as a Stirling motor, was created in 1816 by the Scottish Reverend Robert Stirling and uses a variety of energy sources, including biomass and solar power. Subsequently, Eric Podesser, the first scientist to design and produce a Stirling motor (SE) utilizing biomass, started the use of biomass as fuel for the motor in 1999. Additionally, he proposed a variety of waste materials, such as logs or chopped wood, agricultural waste, fruit shells (e.g., coffee shells) [18]; Podesser and Bayer collaborated in 2000 to assess the Stirling motor performance in an Austrian district heating biomass facility [19].

For biomass-based Stirling systems, most of the research has been conducted on wood; wood powder has been chosen by Akio Nishiyama [20] to test its combustion in a Stirling motor, wood chips have been selected as a fuel to power two types of Stirling motors at the Technical Academy of Denmark (the four-cylinder Stirling and the eight-cylinder Stirling) [21], and wood pellets have been used on a Micro-CHP mechanism to assess its energy performance [22]. In this context, Damirchi et al. [23]. investigated the shape and size of the wood on the power produced by the Stirling to determine the effect of wood type on Stirling Motor power Cardozo et al. assessed the performance of a Stirling motor micro-cogeneration mechanism using bagasse from sugar cane and wood pellets; they concluded that, due to ash accumulation, the efficiency of a Stirling motor cogeneration system should be somewhat reduced when bagasse pellets are used in place of commercial wood pellets [24].Other researchers suggested novel approaches including combining a biomass Stirling motor with an ORC in a micro-CCHP (μ-CCHP) system [25], and they suggested adding a chemical agent like Al_2_O_3_ or Sio_2_ nanofluid to optimize the burning of biomass [26]. In contrast, however, scholars have been drawn to the study of olive cake conversion by the Stirling engine; Rassai et al. presented a study and design of a Stirling engine cogeneration machanism powered by olive cake [28] and examined the effect of olive cake particle size on combustion characteristics in a Moroccan Stirling engine cogeneration system [27]. In order to identify the ideal fuel for a CHP system and the thermodynamic cycle, Najah el idrissi et al. compared the combustion of Moroccan argan nut shell and olive cake [29]. In Ref. [30], they also looked into how the biomass from argan nut shells (ANS) affected the Stirling engine's combustion characteristics.

The aim of this paper is to study the effect of excess air ratio on temperature and emissions, and the impact of olive cake combustion on the environment by comparing the molar concentration of different pollutants such as (CO, CO_2_, NO, NO_2_ …) with limits given by international organizations of health and environment.

## 2 Materials and methods

A. Equations of the problem

Fluid flow, heat and mass movement, and chemical reaction processes are all described by CFD modeling of biomass combustion processes. The fundamental equations for mass, momentum, energy, and species during combustion in a fluidized bed will be covered in this section [31]. We refer to this collection of conservation laws as the Navier-Stokes equations in CFD:

Continuity equation

(1) $$\frac{\partial \rho }{\partial t}+\nabla \left(\rho ū\right)=S_{P}$$

Momentum equation

(2) $$\frac{\partial \left(\rho ū\right)}{\partial t}+\nabla \left(\rho ūū\right)=-\nabla p+\nabla \left(\mu \nabla ū\right)+S_{N}$$

Energy equation

(3) $$\frac{\partial \left(\rho H\right)}{\partial t}+\nabla \left(\rho ūH\right)=\nabla \left(\lambda \nabla T\right)+S_{H\;}$$

Species transport equation

(4) $$\frac{\partial \left(\rho Y_{f}\right)}{\partial t}+\nabla \left(\rho ūY_{f}\right)=\nabla \left(D\nabla \left(\rho Y_{f}\right)\right)+S_{Y}+R_{f}$$

B. Geometry and meshing

The geometry is small scale CHP unit that contains primary and secondary air inlet to control the addition of excess air and the location of the swirl motion (primary, secondary or both) in upcoming studies.

The particle inlets are located perpendicular to the axis of the combustion chamber (CHP unit) to favor the configuration of the fluidized bed and to guarantee a good temperature distribution around the Stirling engine's heater.

**Fig. 1:**
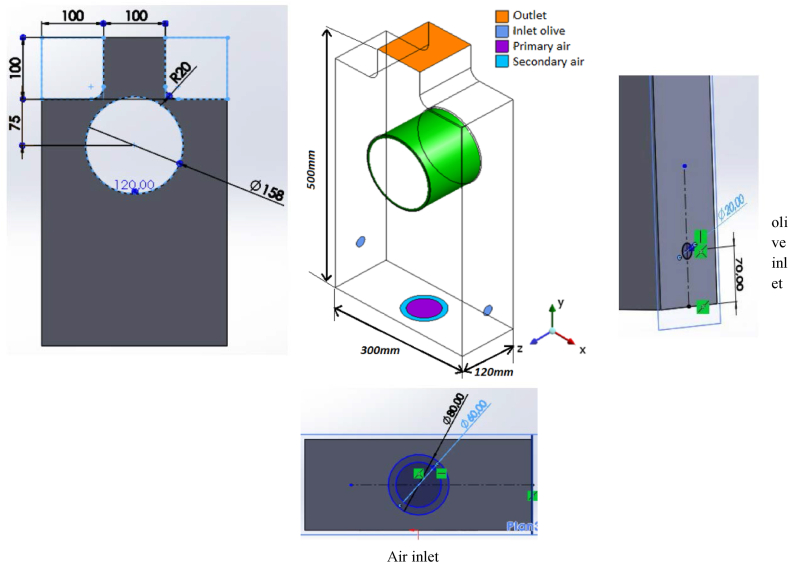
Furnace geometry with the dimensional characteristics.

Fig. 1 illustrates the furnace 3D and 2D view with the dimensional characteristics of the elements constituting it.

Tubes (inlet olive) have a diameter of d1 = 20 mm.

The principal air intake's diameter is measured at d3 = 60 mm.

D4 = 80 mm is the diameter of the secondary air inlet.

The hot heat exchanger of the Stirling motor is the green component.

The maximum number of "hexahedral" elements were generated via mesh building using a cut cell method. To have a better resolution, important areas like olive inlets and outlets are upgraded; there are 943188 components overall. The initial grid was initially coarse (514633 elements) and the convergence criterion was not attained (10^**−4**^ for all elements excluding energy)). Therefore, using face sizing method was necessary to refine critical regions mesh.

**Fig. 2:**
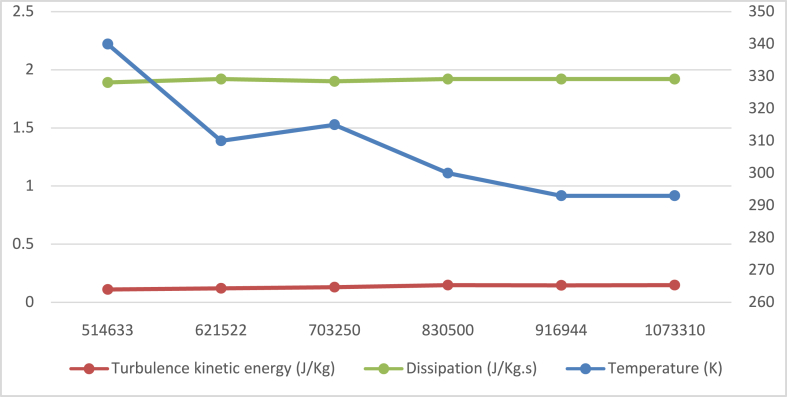
Mesh independence study on the combustor outlet.

**Fig. 3:**
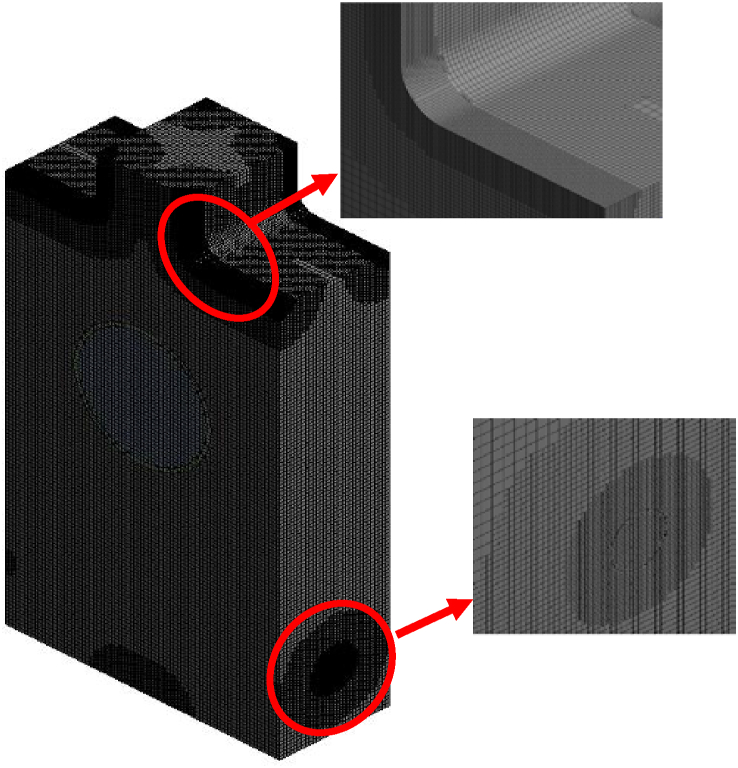
3D generated grid of the furnace.

To ensure the accuracy of the results, a mesh convergence analysis has been conducted. The outlet temperature is calculated for each mesh refinement. Fig. 2 shows the grid independency study results on the furnace outlet for three parameters: turbulence kinetic energy, the dissipation and temperature; the kinetic energy of turbulence and its dissipation [0–2,5] are presented on the primary axis [0–2,5], the temperature is presented on the secondary axis [260−350]. It is evident from this figure that the temperature converges at 9 × 105 cells, yet the turbulent kinetic energy and dissipation converge at 830500 cells. Therefore, this mesh refinement has been applied for the following simulations to be performed during this analysis. 3D mesh details for the whole flow domain of the furnace are depicted in Fig. 3.

Skewness constitutes one of the primary metrics used to assess a mesh's quality. It is based on the parameter. It indicates a face's or cell's proximity to the ideal (equilateral for a triangle, equiangular for a quadrilateral). Skewness defines an equilateral cell (the ideal) as having a value of 0 and a fully debased cell (the weakest) as having a value of 1. The Skewness factor for our mesh is 4.15e^−03^, relating to ANSYS Meshing User's guide; the mesh is excellent because this factor is between 0 and 0.25.

C. Operating conditions

This section is dedicated to the presentation of thermal characteristics of Olive Cake; The proximate and ultimate analysis of olive particle are presented in Table 1, Rosin Rammler parameters in Table 2, and boundary conditions in Table 3.

**Table 1:** Olive cake analysis.

Proximate analysis (wt%, dry)—DAF		Ultimate analysis (wt%, dry)	
Volatile matter	64	Carbon (C)	59
Fixed carbon	23.2	Hydrogen (H)	8.5
Ash	6.5	Nitrogen (N)	1.5
Moisture	6.3	Sulfur (S)	0
		Oxygen (O)	31

**Table 2:** Parameters of olive cake particle.

Parameters	Particle
***Minimum diameter (*μm*)***	20
***Maximum diameter μm)***	100
***Mean diameter (μm)***	70
***Spread parameter***	3.5

**Table 3:** Boundary conditions.

Boundary	Type	Temperature (k)	Mass flow (g/s)
OC inlet	Mass flow inlet	300	0.17
Primary inlet	Mass flow inlet	300	0.91988
Secondary inlet	Mass flow inlet	300	0. 4941
Outlet	Pressure outlet	300	0
Interface furnace-heater	Wall	300	0
Wall furnace	Wall	300	0
Wall heater	Wall	300	0

The particle size distribution is modeled by the Rosin Rammler distribution (Weibull distribution) because it is the more convenient mathematical model of continuous probability distribution used to describe pulverized particle size distributions generated by milling, grinding, and crushing operations. A sufficient number of discrete intervals are created from the full-size range, and each is represented by an average diameter when trajectories computations are carried out. The injected particle has a diameter of 70 μm (Table 2).

'The proximate and ultimate analysis of olive particle is taken from Elorf et al. (2016) [17] (Table 1), the analysis followed ASTM standars; The ultimate analyzer was used to burn the sample of olive particles in order to determine the weight of carbon, nitrogen, hydrogen, sulfur, and ash.

Table 3 shows boundary conditions of the simulation with the mass flow of Olive Cake and air; in the furnace, the combustion process functions with different excess coefficients (λ_1_ = 1.1, λ_2_ = 1.3, λ_3_ = 1.57) at atmospheric pressure.

D. Numerical method

The computational model runs under the software ANSYS 14.5 and computes the movement equations using the finite volume method. The flow inside the furnace is regarded as constant, incompressible, and turbulent, and a solution is found using the Navier-Stokes pressure-based solution algorithm.

For the species model, the empirical fuel stream and the coal calculator option for non-premixed combustion were used. Non-premixed simulation entails calculating transport equations for a couple of conserved balances (mixing percentages). The species-specific equations remain unsolved. Consequently, the predicted mixing fraction fields are used to compute the species concentrations. Preprocessing and compilation of the thermochemistry calculations are done in ANSYS FLUENT for research purposes. The chemistry and turbulence interact with the help of a probability density function (PDF) with an assumed shape. The solution was calculated using the pressure-based Navier-Stokes algorithm; the pressure-velocity coupling was handled by the SIMPLE algorithm; the combustion process was carried out using the non-premixed model (PDF mixing fraction); the radiation was handled by the P1 model; the particle phase was handled by the discrete second phase; the turbulence was handled by the k-ε feasible model with thermal effects in the enhanced wall treatment; and the coal combustion was handled by the diffusion-limited coal combustion model (DLCCM).

A mixing problem simplifies combustion and eliminates issues with closing the non-linear average reaction velocities. Using the Equilibrium model, the chemistry can be represented as being in chemical equilibrium once combined. Twenty species (CO, CO_2_, C, C(s), H, H_2_, O, O_2_, N, N_2_, OH, H_2_O, C_2_H_2_, CH_4_, CH_3_, C_2_H_4_, HO_2_, C_2_H_6_, H_2_O(l), and HCN) make up the PDF calculated equilibrium system for the firing of olive cake.

Primary air has a Reynolds number of 14474, while secondary air has a number of 3331. The verification of the turbulence and burning model has been detailed in the previous paper by Rassai et al. [27], the results of CFD are verified with the measured results from experimental data, and the study reveals firstly that the RKE model is the more accurate in comparison with other models (Standard and RNG) and secondly that the non-premixed combustion model gives satisfactory outcomes for emissions (CO_2_, CO, H_2_O, and O_2_) and gas temperature. Therefore, our numerical model is valid, and we can use it for this study to model the gas combustion phenomena.

E. Modeling multiphase

Three computing methods can be used to represent two-phase fluid flow: discrete element method (DEM-CFD) within the Eulerian-Lagrangian idea, Eulerian-Eulerian, and Eulerian-Lagrangian with a single particle or a particle packet and a particle group [31].

The multiphase flow was modeled here using the Euler-Lagrangian method, where the discrete phase is solved in a Lagrangian frame of reference by following a large number of bubbles, particles, or droplets through the calculated flow field, and the main phase is seen as a continuum phase by solving the time-averaged Navier Stokes equations. This method allows for the exchange of mass, momentum, and energy between distributed and main phases. This model's fundamental premise is that, even though the scattered secondary phase's mass may exceed that of the main phase, its volume fraction will be less than 10–12% [15].

The track of a particle in discrete phase is estimated by calculating the total forces on the particle [32].

(5) [φξ9]

Where $FD\left(\vec{u}+\vec{u}p\right)$ where indicates the drag force value per particle mass unit. defines the drag force per unit mass of particle.

The expression of *F*_*D*_:

(6) $$FD=\frac{18\mu }{\rho_{p}D_{P}^{2}}\frac{C_{D}{\text{Re}}_{p}}{24}$$

and

(7) $$R_{e_{p}}=\frac{\rho D_{p}|u_{p}-u|}{\mu }$$

R_ep_ represents the relative Reynolds number, while C_D_ is the drag coefficient.

## 3 Results and discussions

This study has two objectives; the first is examining the impact of excess air ratio on temperature profile and emissions, the second is investigating the impact of olive cake combustion on the environment by comparing the molar concentration of different pollutants such as (CO, CO_2_, NO, CH_4_ …) with limits given by international organizations of health and environment.

1. Effect of excess air coefficient

Among parameters influencing the firing process and the emission of pollutants there is the excess air coefficient (λ) or the equivalence ration (Φ = 1/λ), λ is the actual air divided by the stoichiometric air. To well understand the process of the combustion and to minimize greenhouse gases, a study of the effect of excessive air is necessary. Therefore, this part is consecrated to the investigation of the effect of three excess air coefficients on main parameters: temperature, streamline and species at the outlet of the furnace. And that, to control the combustion process, to choose the most appropriate excess air coefficient, to better understand the different mechanisms that take place in the burning systems of olive cake and to anticipate any problems that may occur.

Three excess air coefficients (λ_1_ = 1.1, λ_2_ = 1.3, λ_3_ = 1.57) are chosen in order to examine the effects of extra air coefficient on temperature profiles and the emissions at the outlet of the furnace (line y/z = 10.33).

A. Effects on the temperature

**Fig. 4:**
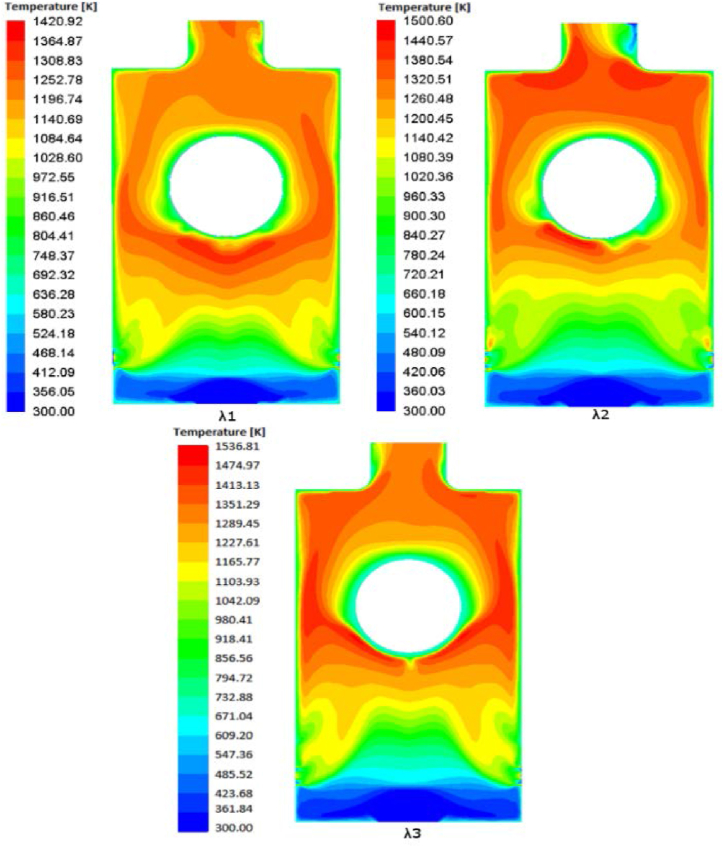
Contours of temperature for λ_1_ = 1.1, λ_2_ = 1.3, λ_3_ = 1.57.

The temperature contours for the three surplus air ratios (λ1, λ2, and λ3) are displayed in Fig. 4, and it is evident that the contour geometries differ for each excess air ratio. The absence of oxygen at the outflow may be the reason for the nearly symmetrical temperature profiles except for λ2. The highest temperature for λ1 is 1420.92 K, the highest temperature for λ2 is 1506.60 K, and the highest temperature for λ3 is 1536.81 K; this demonstrates that as the excess air ratio increased, the temperature value increased. This can be explained by the existence of sufficient amount of air in case three (λ_3_) which leads to complete combustion of Olive Cake. Moreover, for λ_1_ and λ_2_ cases, the temperature is not well distributed around the heater and the higher temperatures are on above the heater near to the outlet. While for the λ_3_ case, the temperature is well dispersed in the combustor which will lead to a good temperature distribution over the heater.

B. Influence on emissions

**Fig. 5:**
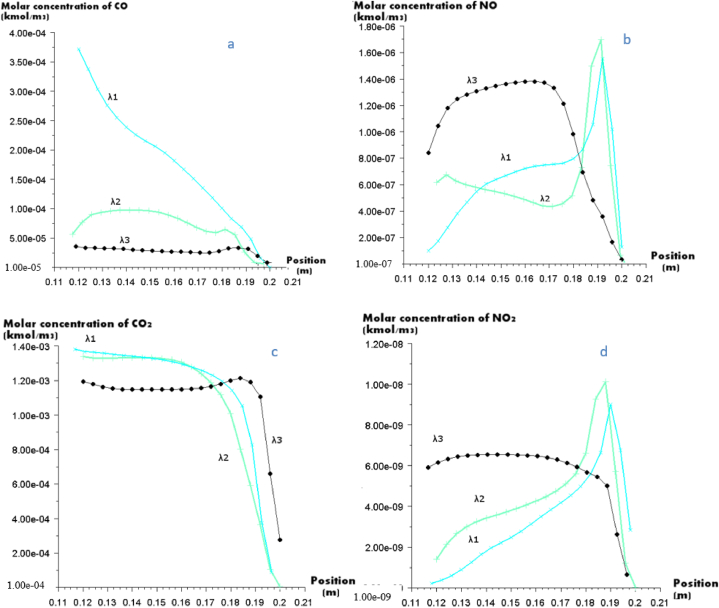
(a). Molar concentration of CO. (b) Molar concentration of NO. (c) Molar concentration of CO_2_, (d) Molar concentration of NO_2_ at the outlet of the furnace for three excess air coefficients.

**Fig. 6:**
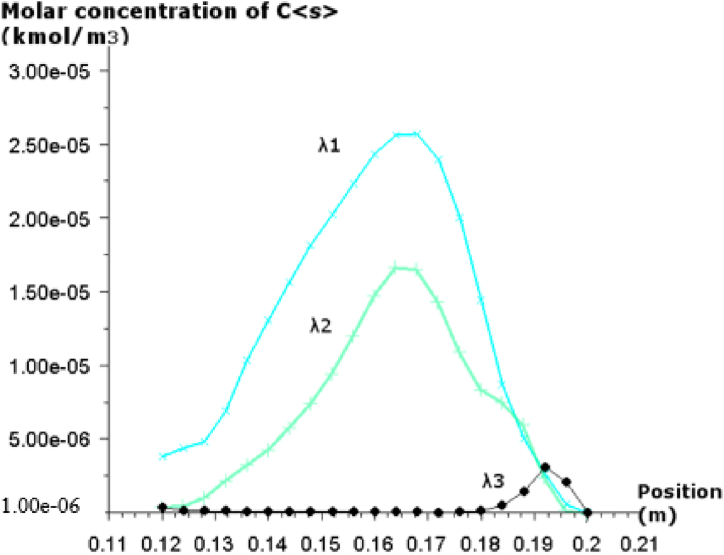
Molar concentration of C <s> at the outlet of the furnace for three excess air coefficients.

Fig. 5, Fig. 6 depict the variation in the molecular concentrations of NO, NO_2_, CO, CO_2_, and C<s> in relation to the excess air ratio during the burning of olive cakes, respectively.

Fig. 5 (a) illustrates the effects of the ratio of surplus air on CO emissions at the outlet of the furnace for Olive Cake combustion. CO concentration decreases as the surplus air coefficient increases from 1.1 to 1.57. While the maximum CO emission is 3.75e^−04^ kmol/m^3^ at λ_1_ = 1.1, it becomes 2.75e^−05^ kmol/m^3^ at λ_1_ = 1.57 due to the existence of O_2_. It is clear from Fig. 5 that there is a negligible difference of CO_2_ molar concentration for the three excess air ratios (1.4e^−03^ for λ_1_ and λ_2_; 1.2e^−03^ for λ_3_). In addition, plants absorb CO_2_ during photosynthesis, a process that lasts the entirety of a plant's life, when biomass sources are burned. Consequently, during burning, CO_2_ that has been taken up from the atmosphere by photosynthesis is returned to it. Olive Cake does not contribute any net amount of CO_2_ to the atmosphere.

Fig. 5(b–d) shows that the molar concentration of NO and NO_2_ increase as the rate of excess coefficient increases except at the region between 0.18 and 0.192. This increase may indicate that better nitrogen combustion has been achieved, particularly in the freeboard area, as a result of the increased O2 supply, which raised the combustion temperature and increased the production of NOx.

Fig. 6 presents the C<s> molar concentration at the outlet of the furnace for the three excess coefficient ratio. It can be noticed that the molar concentration of C<s> decreases as the excess ration increase, and limited quantity is formed when λ_3_ is more than 30%. These observations can be explained by the good mixing of particles with air, especially when there is an important excess air and also having a long residence time of the particles in the combustion zone.

According to this study, an increase in the excess air ratio causes generally a complete combustion and then higher combustion efficiency due to the formation of few quantities of CO and C<s>. Additionally, the rise of excess air ratio causes temperature rise in the furnace and generates a well temperature distribution contours in the furnace and around the heater. The results of this investigation show also that λ_3_ = 1.57 gives the minimum emissions and needs minimum calculating time of OC combustion process.

2. Environmental impacts of olive cake combustion

As a tool for global development, the MDGs, or Millennium Development Goals, expire in 2015. Consequently, the post-2015 agenda is the center of attention for the international community. As a result, the global community's attention is focused on the post-2015 agenda, which emphasizes the green economy. Sustainable development is viewed as an element of environmentally friendly development that can assist in achieving particular goals and quantifiable enhancements at the junction of the economy and environment Waste materials is viewed in this perspective as a significant economic burden in addition to an environmental problem [33]; the environmental problem of solid waste depends on the composition of the waste and the method of its recovery (fermentation, combustion …).

For this purpose, an environmental impact study of our olive cake combustion is required to quantify the pollutant emissions to the air and compare them with guideline values given by the World Health Organization (WHO); molar concentration of the primary contaminants of olive cake combustion in different verticals positions will be presented and discussed in this part.

**Fig. 7:**
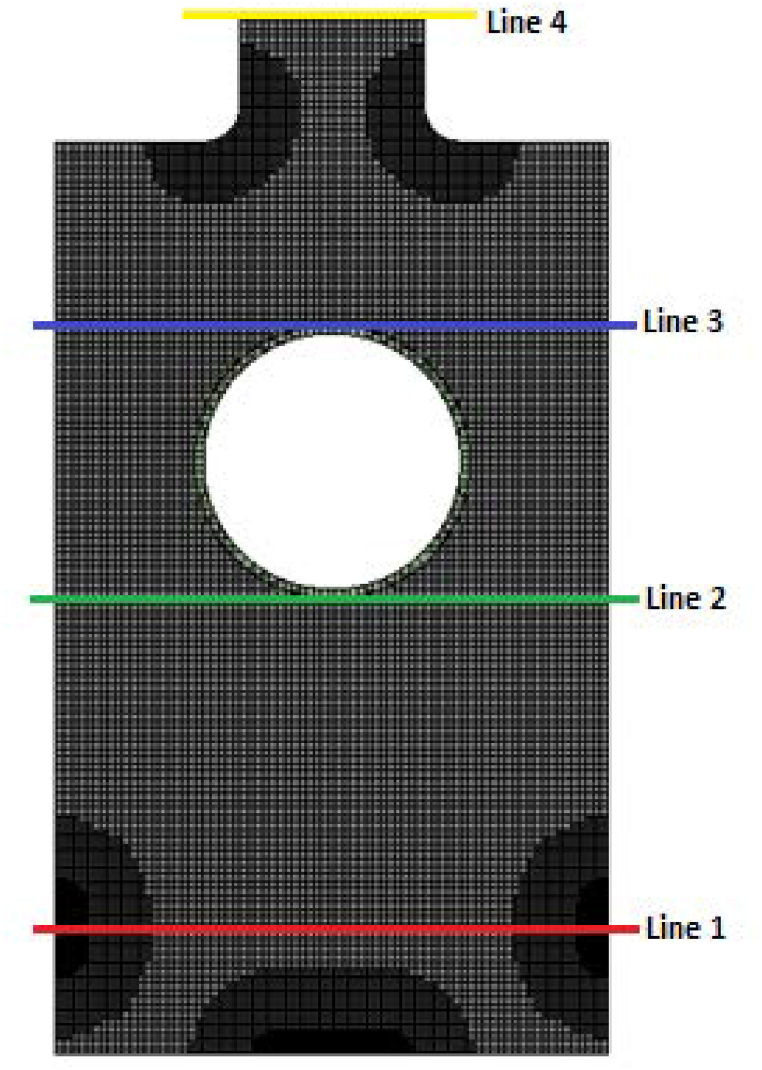
Different vertical positions on the furnace.

Fig. 7 shows the different vertical positions on the combustor chamber; line 1 (y/z = 1.16), line 2 (y/z = 3.75), line 3 (y/z = 6.66), line 4 (y/z = 10.33). The discussions will be focused on line 4 because it is the interface between the furnace and ambient air.

According to WHO, the main pollutants of air that have dangerous consequences on human health are Ozone (O_3_), Particulate Matter (PM), Sulfur Dioxide (SO_2_), Nitrogen dioxide (NO_2_); They may aggravate ocular irritation, aggravate asthma, impair lung function, and result in lung disorders. For this reason, WHO creates and publishes air quality guidelines [34]. on another hand, the emissions of our olive cake combustion don't contain particulate matter (PM), sulfur dioxide (SO_2_), and ozone (O_3_) because the olive cake is free from sulfur. Therefore, the combustion of olive cake has just one pollutant (NO_2_) that may have impacts on human health contrary to other biomass that emitted these dangerous pollutants.

**Fig. 8:**
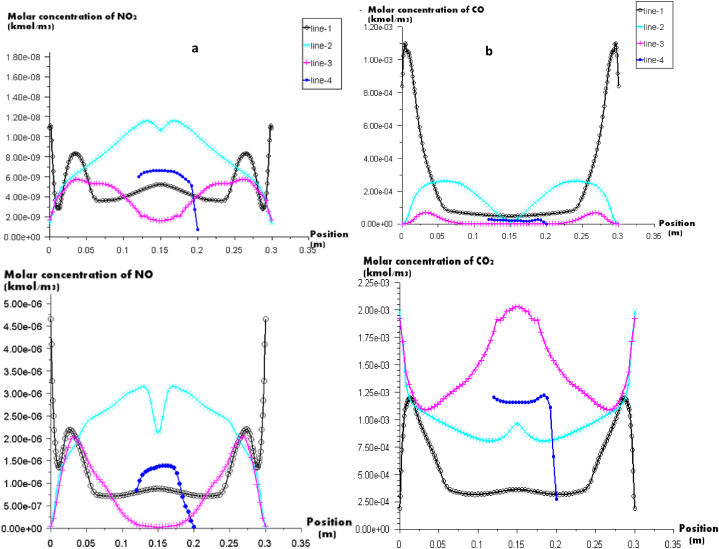
(a) Molar concentration of NO and NO_2_. (b) Molar concentration of CO and CO_2_ in different lines of the furnace.

Fig. 8 (a) illustrates the molar concentration of NO_2_ and NO on different vertical positions on the furnace. It shows that the highest values of the molar concentration of NO_2_ are found on line 2 under the heater; the maximum value reaches 1.2e^−08^ kmol/m^3^ on the medium of the furnace (x = 0.125 m and x = 0.212 m). Moreover, line 1 presents high values of NO_2_ at the olive inlet (x = 0 m and x = 0.3 m), this increase in molar concentration of NO_2_ can be explained by the reaction of NO and O_2_ in these areas.

Concerning line 4 that presents the contact of the furnace with the ambient air, the maximum value of the molar concentration of NO_2_ is 6e^−09^ kmol/m^3^ (the equivalent of 276 μg/m^3^). A guideline value of 200 μg/m3 (hour mean) has been set by the World Health Organization (WHO) [35] to safeguard the public against the harmful effects of this gas on health. The combustion of olive cake provides a higher value than that fixed by WHO (difference of 76 μg/m^3^). This indicates that more research is needed to determine how to lower NO2 emissions in order to abide by WHO guidelines and safeguard public health. In addition, the maximum values of the mass fraction of NO are located near the burner inlet (line 1) and under the heater (line 3) at x = 0.1 m and x = 0.2 m; the trend of NO molar concentration starts increasing until achieving 3.5.10^−4^ at x = 0.125 m and starts decreasing until reaching 0 at x = 0.15. After that, it increases for the second time. This increase and decrease can be explained by the change in temperature profile due to the fact that when the flame temperature is, the NO mass fraction is. The mass fraction of NO emitted on air is lower in comparison with the mass fraction emitted inside the furnace; it is about 1,3.10^−4^ kmol/m^3^ (the equivalent of 39 mg/m^3^). This means that the combustion of olive cake will not emit high quantities of pollutant NO on air, and then doesn't degrade the air quality. In addition, no value has been fixed by the WHO not to exceed, but some research set the emission limit value from 1900 mg/m^3^ to 4750 mg/m^3^ for combustion plants and boilers [36].

Fig. 8(b) presents the profiles of the molar concentration of CO and CO_2_ in different vertical positions. The CO reaches the maximum in line 1 near to the burner inlet. This can be explained by the presence of the combustion process at the inlet and the insufficiency of O_2_ to produce CO_2_. And because carbon monoxide has a dangerous effect on human health; It prevents the blood from supplying essential organs like the heart and brain with oxygen, the World Health Organization has set a maximum value of the molar concentration of CO at 30000 μg/m^3^ [34]. Additionally, the maximum allowable CO concentration for exposure has been set at 35000 μg/m^3^ by the Occupational Safety and Health Administration (OSHA) [34]. On the other hand, it can be seen in the figure that line 4 is characterized by lower values of CO (about 0.25 × 10^−04^ kmol/m^3^); it is the equivalent of 7000 μg/m^3^, this quantity is less than those fixed by WHO and OSHA which means that the combustion doesn't contribute to the degradation of the air quality.

When it comes to CO_2_, the highest mass fraction values are found close to the burner exit (line 3), and CO_2_max = 2.5 × 10-03 kmol/m3 is the value of this molar concentration of CO_2_. In contrast to the other lines, this one produces CO_2_ much more quickly since it is mostly composed of CO_2_ from devolatilization in this area and CO_2_ that is produced through oxidation of CO. In other sections, the CO_2_ is lower due to the devolatilization. Also, these sections are characterized by the presence of high quantity of CO. The line 4 represents the quantity of CO_2_ emitted by the burning of olive cake to the ambient air; it is approximately of 1.2 × 10^−3^ kmol/m^3^, which is the equivalent of 52,821 ppm, that do not exceed the value fixed by American Society of Heating, Refrigerating and Air conditioning Motoreries (ASHRAE) and Occupational Safety and Health Administration (OSHA); 1000 ppm [37].

**Fig. 9:**
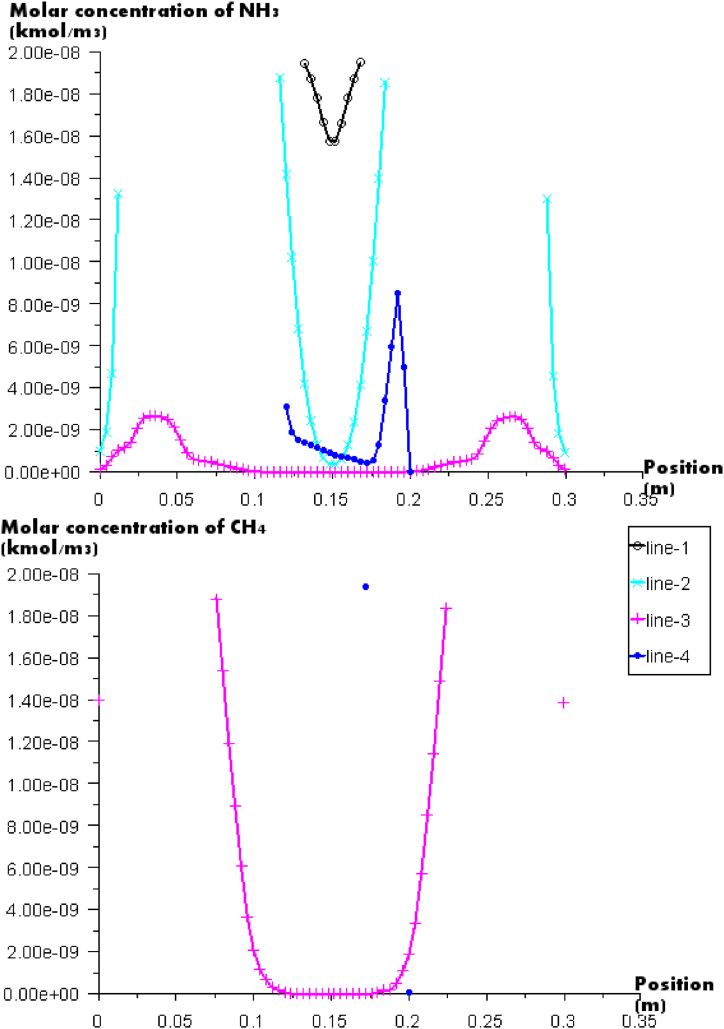
Molar concentration of NH_3_ and CH_4_ in different lines of the furnace.

Fig. 9 presents molar concentration of NH_3_ and λ_1_ in different vertical position; NH_3_ is a toxic gas that causes respiratory damage and death. It is clear that the CH_4_ is a hydrocarbon that is a greenhouse gas with a global warming potential [38]; it is one of the dangerous gases that contributes to global warming. For that it is taken into account by Directive 2003/87/EC. In another hand, The National Institute for Occupational Safety and Health (NIOSH) set the maximum level of methane for workers during an 8-h workday at 1000 ppm (0.1 percent), but the Occupational Safety and Health Administration (OSHA) has not established a permissible exposure limit for the gas [39]. It is clear from Fig. 9 that CH_4_ is not present at the outlet of the furnace; just one point in the medium that presents 32 0.10^−5^ ppm; this value is negligible compared to the value given by NIOSH. This result can be explained by the absence of recirculation zones in this line and the decrease of the temperature [1227,45K-1289,61K] due to the position of the heater.

## 4 Conclusion

The purpose of this paper was to investigate the effect of surplus air ratio on several burning parameters of olive cake like temperature and emission. In this context, turbulence, combustion of gas phase and particle phase are simulated under ANSYS Fluent Software.

In conclusion, the excess air ratio λ_3_ = 1.57 is more appropriate for this case because the given gas temperature is higher than that provided by λ_1_, λ_2_. In addition, the mass fraction of CO, CO_2_, and C char was lower; this finding demonstrates that the emission of greenhouse gases was influenced by the excess air ratio even if we had the same biomass and content. On the other hand, the presence of low amounts of C char in this study is important because the existence of unburned substants can create problems for the operation and maintenance of the Stirling motor, in particular for the heater.

The second part of this study investigates the presence of pollutant species in different vertical attitudes of the furnace. It can be concluded that the emissions are lower at the outlet of the furnace and are within the guideline fixed by some organizations such as The World Health Organization (WHO), the Occupational Safety and Health Administration (OSHA), and the National Institute for Occupational Safety and Health's (NIOSH). Therefore, the combustion of olive cake with these selected parameters doesn't affect the quality of air and doesn't present any danger to human health and the environment. Moreover, λ_3_ has another advantage compared to other excess air ratio; it needs minimum calculating time (about 72 h). All these advantages encourage investigating the power generated by Olive Cake with the selected parameters within a Stirling cogeneration system in the further research.

## Funding statement

This research did not receive any specific grant from funding agencies in the public, commercial, or not-for-profit sectors.

## Data availability statement

No data was used for the research described in the article.

## CRediT authorship contribution statement

**N. Rassai:** Writing – review & editing, Writing – original draft, Visualization, Validation, Software, Methodology, Formal analysis, Data curation, Conceptualization. **N. Boutammachte:** Writing – review & editing, Supervision.

## Declaration of competing interest

The authors declare that they have no known competing financial interests or personal relationships that could have appeared to influence the work reported in this paper.

## References

[1] Inauen S. (2017).

[2] (2019). Amisy machinery. https://www.wood-pellet-mill.com/.

[3] (2019). Energie. https://www.usinenouvelle.com/.

[4] (2020). Annual financial report. https://www.cosumar.co.ma/.

[5] Lafargeholcim, https://www.lafargeholcim.com/, [accessed 11 august, 2022].

[6] Veolia reports, https://www.veolia.com//, [accessed 15 august, 2022].

[7] Agrimaroc news , https://www.agrimaroc.ma//,[accessed 15 august, 2022].

[8] Aveo , http://www.aveo.ma/, [accessed 15 august, 2022].

[9] (2019). International olive council database. http://www.internationaloliveoil.org/.

[10] Hüseyin T., Aysel T.A., Ali D. (2003). Olive cake combustion in a circulating fluidized bed. Fuel.

[11] Kuosa M., Kaikko J., Koskelainen L. (2007). The impact of heat exchanger fouling on the optimum operation and maintenance of the Stirling engine. Appl. Therm. Eng..

[12] K. Lyk, K. Andersen, «CFD Biomass Combustion– Present Applications», Force Technology.

[13] Mehrabian R., Scharler R., Obernberger I. (2013).

[14] Mehrabian R., Shiehnejadhesar A., Scharler R., Obernberger I. (2014). Multi-physicmodelling of packed bed biomass combustion. Fuel.

[15] Singh R.I., Brink A., Hupa M. (2013). CFD modeling to study fluidized bed combustion and gasification. Appl. Therm. Eng..

[16] Bonefacic I., Frankovic B., Kazagic A. (2015). Cylindrical particle modelling in pulverized coal and biomass co-firing process. Appl. Therm. Eng..

[17] Elorf A., Koched N., Boushaki T., Sarh B., Chaoufi J., Bostyn S., Gökalp I. (1951-1971, dec. 2016). Swirl motion effects on flame dynamic of pulverized olive cake in a vertical furnace. Combust. Sci. Technol..

[18] Podesser Erich (1999). Electricity production in rural villages with a biomass Stirling engine, Renew. Energy.

[19] Podesser E., Bayer H. (2000). Application and economy of biomass Stirling engines in Austria. World Renewable Energy Congress VI (WREC).

[20] Nishiyama A., Shimojima H., Ishikawa A., Itaya Y., Kambara S., Moritomi H., Mori S. (2007). Fuel and emissions properties of Stirling engine operated with wood powder. Fuel.

[21] Stirling engines for biomass applications; sustainable power production; stirling danmark diplomvej DTU, Building 373 South DK-2800 Lyngby Denmark.

[22] Voronca S., Siroux M., Darie G., Kallio S. (2022). Simulating ON-OFF regimes on a micro-CHP using biomass. Earth and Environmental Science.

[23] Damirchi H., Najafi G., Alizadehnia S., Ghobadian B., Yusaf T., Mamatd R. (2015). Design, fabrication and evaluation of gamma-type stirling engine to produce electricity from biomass for the micro-CHP system. Energy Proc..

[24] Cardozo E., Malmquist A. (2019). Performance comparison between the use of wood and sugarcane bagasse pellets in a Stirling engine micro-CHP system. Appl. Therm. Eng..

[25] Udeh G.T., Michailos S., Ingham D., Hughes K.J., Ma L., Pourkashanian M. (2021). «A techno-enviro-economic assessment of a biomass fuelled micro-CCHP driven by a hybrid Stirling and ORC engine. Energy Convers. Manag..

[26] Najafi G., Hoseini S.S., De Goey L.P.H., Yusaf T. (2020). Optimization of combustion in micro combined heat and power (mCHP) system with the biomass-Stirling engine using SiO2 and Al2O3 nanofluids. Appl. Therm. Eng..

[27] Rassai N., Boutammachte N., El hassani H., Almers A., Boudi E.M. (2018). Bekraoui, « Effect of the particle size of pulverized olive cake on combustion parameters in Stirling engine in Morocco. Case Stud. Therm. Eng..

[28] N. Rassai, A. Najah elidrissi , W Ayrir, N. Boutammachte, « Study and design of Stirling motor cogeneration system powered by olive cake.», International Review of Mechanical Engineering (I.RE.M.E.), vol. 17, n. 4.

[29] Najah EL idrissi A., Benbrahim M., Rassai N. (2023).

[30] Najah EL idrissi A., Benbrahim M., Rassai N. (2023). «Effect of the particle size argan nut shell (ANS) biomass on combustion parameters in stirling engine in Morocco. Results in Engineering.

[31] Shi A., Pang Y., Xu G., Li C. (2015). Numerical simulation of biomass gasification in a fluidized bed. International Conference on Applied Science and Engineering Innovation.

[32] Singh R., Brink A., Hupa M. (2013). CFD modeling to study fluidized bed combustion and gasification. Appl. Therm. Eng..

[33] Korytnyi E., Saveliev R., Perelman M., Chudnovsky B., Bar-Ziv E. (2009). Computational fluid dynamic simulations of coal-fired utility boilers: an engineering tool. Fuel.

[34] Sanusi Y.A. (2010). Capacity issues of private sector participation in urban solid waste management in Nigeria. Humanity and Social Sciences Journal Pakistan.

[35] who. https://www.who.int/.

[36] Emissions. https://sites.google.com/site/emissionatmospherique//.

[37] OMS reglementation. https://www.airparif.asso.fr//.

[38] Engineering. https://www.engineeringtoolbox.com/.

[39] Atta Atia, PhD (2004).

